# Analysing the In-Use Stability of mRNA-LNP COVID-19 Vaccines Comirnaty™ (Pfizer) and Spikevax™ (Moderna): A Comparative Study of the Particulate

**DOI:** 10.3390/vaccines11111635

**Published:** 2023-10-25

**Authors:** Jesús Hermosilla, Airan Alonso-García, Antonio Salmerón-García, José Cabeza-Barrera, Antonio L. Medina-Castillo, Raquel Pérez-Robles, Natalia Navas

**Affiliations:** 1 Department of Analytical Chemistry, Science Faculty, University of Granada, Fuentenueva Avenue, 18071 Granada, Spain; jesushf@ugr.es (J.H.); antonioluismedina@ugr.es (A.L.M.-C.); raquelpr@ugr.es (R.P.-R.); 2 Instituto de Investigación Biosanitaria de Granada (ibs.GRANADA), 18012 Granada, Spain; antonio.salmeron.sspa@juntadeandalucia.es (A.S.-G.);; 3 Department of Clinical Pharmacy, San Cecilio University Hospital, Conocimiento Avenue, 18016 Granada, Spain; 4 Fundación para la Investigación Biosanitaria de Andalucía Oriental-Alejandro Otero, Madrid Avenue, 18012 Granada, Spain

**Keywords:** COVID-19 vaccines, Comirnaty™, Spikevax™, DLS, in-use stability testing, TEM

## Abstract

Comirnaty™ and Spikevax™ were the first vaccines approved for human use based on modified non-replicating mRNA lipophilic nanoparticle (mRNA-LNP) technology, with great success in the treatment of COVID-19. They have been used massively worldwide. One of the major inconveniences of these vaccines is related to pharmaceutical stability issues. Proper transportation, storage, and in-use handling before administration to patients are critical steps since failures can potentially reduce potency. In this research, the in-use stability of Comirnaty™ and Spikevax™ clinical samples was analysed and the results were compared. As changes in the size of the mRNA-LNPs are related to potency, these modifications were analysed by qualitative Dynamic Light Scattering (DLS) as a stability-indicating method for control and stressed vaccine samples. Strong stress factors (accelerated light irradiation, manual shaking, and vortex vibration) and conditions that mimic in-use handling (exposure to natural light and room temperature, repeated cycles of injections, and 24 h storage in syringes) were checked. The morphology of the mRNA-LNPs was analysed by Transmission Electron Microscopy (TEM) to better interpret and support the DLS results. Although the two vaccines are based on the same mRNA-LNP technology, the results demonstrate that they are characterised by very different particle size profiles and behaviours against different handling/stress conditions.

## 1. Introduction

The recent pandemic caused by the SARS-CoV2 virus has revealed a new vaccination strategy based on the encapsulation of the mRNA into lipid nanoparticles (mRNA-LNPs) [1]. This has allowed for a rapid, safe, and effective response from the pharmaceutical field (public/private sector collaboration) for the COVID-19 pandemic, declared on 11 March 2020 by the World Health Organization (WHO). Recently (5 May 2023), the WHO determined that “COVID-19 is now an established and ongoing health issue which no longer constitutes a public health emergency of international concern” [2]. Notwithstanding the latter, it cannot be lowered the guard against this disease, and the WHO updated the 2023–2025 COVID-19 Strategic Preparedness and Response Plan [3]. Vaccine research is one of the 10 COVID-19 operational pillars proposed in this document, and WHO’s role in supporting Member States’ vaccination programmes will continue. 

Comirnaty™ and Spikevax™ are the first vaccines approved for human use using this new delivery strategy based on mRNA-LNPs that encapsulate modified non-replicating mRNA lipophilic nanoparticles (mRNA-LNP), vaccines in which the nucleic acid encodes for the Spike protein [4]. They have been the most administered, for example, in Europe [5]. Therefore, this nanotechnology has proved successful in the prevention of COVID-19, demonstrated to be a fast and secure platform, leading to the development of future mRNA-LNP-based products [6]. However, one of the major inconveniences of these types of vaccines is related to pharmaceutical stability issues [7]. Proper transportation, storage, and in-use handling before administration to patients are critical steps since failures can potentially reduce potency, thereby causing problems in the immune response. New products with improved in-use stability and storage profiles are required to meet medical needs worldwide [8]. In addition, the published literature regarding the long-term stability of mRNA-LNPs is scarce [4], likely attributed to the great emergency generated by the COVID-19 pandemic that promoted fast authorisation by regulatory authorities worldwide.

The stability of these two vaccines is deeply related to temperature, which limits its global use by ultracold storage requirements [9]. Nevertheless, and despite being based on the same nanotechnology, they have different thermal stabilities [10], likely related to the different LNP composition, both in the components and in the molar lipid ratios [4]. Comirnaty™ (tozinameran) is a concentrate for dispersion for the injection of the COVID-19 mRNA vaccine (nucleoside modified) [11]. Each purple vial contains 0.45 mL of white to off-white concentrate, which is diluted in 1.8 mL of 0.9% sodium chloride solution to give a total volume of 2.25 mL (six doses). The long-term stability of the frozen dispersion is 18 months stored at −90 °C to −60 °C; for transportation, vials can be stored at −25 °C to 15 °C for a single period of two weeks and can be returned to the previous temperature stored conditions. Thawed vials are stable for one month at 2 °C to 8 °C but keep this stability for only 48 h when transported, and these unopened vials remain stable for up to two hours at 30 °C prior to use. Once the dilution has been performed, chemical and physical in-use stability has been demonstrated for up to 6 h at 2 °C to 30 °C. Spikevax™ (elasomeran) is a white to off-white dispersion for injection with different formats: 0.2 mg/mL multidose vials (red flip-off cap) with a maximum of 10 doses (50 mcg per dose) and 20 doses (25 mcg per dose); and 0.1 mg/mL multidose vials (blue flip-off cap) containing five doses of 50 mcg and pre-filled syringes with individual doses of 0.5 mL (50 mcg). The long-term stability of the frozen dispersions is 9 months stored at −50 °C to −15 °C. Chemical and physical in-use stability has been proved for the unopened vial stored at 2 °C to 8 °C protected from light for up to 30 days once thawed; within this period, up to 12 h may be used for transportation at the same temperature conditions. Chemical and physical in-use stability has been demonstrated for 19 h at 2 °C to 25 °C after the initial puncture (within the allowed use period of 30 days at 2 °C to 8 °C and including 24 h at 8 °C to 25 °C).

Few research groups have assessed the in-use stability of the Comirnaty™ vaccine. To the best of our knowledge, among the published papers, none have focused on the Spikevax™ vaccine. The colloidal stability of the Comirnaty™ vaccine in syringes made of different materials, when subjected to mechanical—manually pumped through 2 mL syringe and leftovers in vials vortexed—and thermal—25 °C/24 h and 40 °C/1 h—stresses of relatively low intensity and short duration was demonstrated [12]. Notwithstanding this, knocking over or dropping Comirnaty™ vaccine samples from small heights showed a low level of physical instability, indicating a low risk of compromising efficacy [13]. Also, changes in size and species distributions of Comirnaty™ samples after exposure to different stress factors (heat −30 °C, 50 °C and 80 °C for 4 h, mechanical, vortexed, and freeze/thaw stress) in PBS buffer have been reported [14].

In this research, the in-use stability of Comirnaty™ and Spikevax™ clinical samples was analysed and results compared. For this purpose, modifications of the size and structure of the mRNA-LNPs that contain the mRNA were qualitatively analysed by Dynamic Light Scattering (DLS) as a stability-indicating method for control and stressed vaccines samples. Strong stress factors (accelerated light irradiation, manual shaking, and vortex vibration) and conditions that mimic in-use handling (exposure to natural light and room temperature, repeated cycles of injections and 24 h storage in syringes) were investigated. The morphology of the mRNA-LNPs was analysed by Transmission Electron Microscopy (TEM) to better interpret and support the DLS results. To the best of our knowledge, this is the first work showing this kind of data on the Spikevax™ vaccine.

## 2. Materials and Methods

### 2.1. Vaccines, Solutions, and Materials

To prepare the clinical dispersions of the vaccines, unexpired and expired multidose vials from different batches of Comirnaty™ (Pfizer-BioNTech: Pfizer Manufacturing Belgium NV, Puurs, Belgium and BioNTech Manufacturing GmbH, Mainz, Germany) and Spikevax™ (Moderna: Moderna Biotech Spain S.L., Madrid, Spain) (Table 1), 10 mL sodium chloride 0.9% IV injection ampoules (B. Braun, Melsungen, Germany), BD Microlance™ 3 18 G x 1 ½″ needles, 1 mL U-100 Insulin BD Plastipak™ syringes, and 5 mL syringes (Becton, Dickinson and Company Limited, Drogheda, Ireland) were used. All these materials were supplied by the Hospital Pharmacy Unit from the San Cecilio University Clinical Hospital (Granada, Spain). For stress testing, 2 mL amber RAM vials with a 9 mm thread and 2 mL clear RAM vials with a 9 mm thread (Symta, Madrid, Spain) were used.

### 2.2. Stability Study

#### 2.2.1. Aseptic Pharmaceutical Preparation

The unexpired and expired Comirnaty™ and Spikevax™ vials were stored refrigerated at 2–8 °C until use. The clinical dispersions were handled and prepared according to their respective summaries of product characteristics [11,15]. Accordingly, Comirnaty™ original multidose vials (purple tape; 0.45 mL of prediluted concentrate) were diluted in NaCl 0.9% as follows: 1.8 mL of saline solution was taken from the NaCl ampoules and injected into the vials to result in a total volume of 2.25 mL (6 doses of 0.3 mL). In the case of Spikevax™, the original multidose vials (red tape; 5 mL) contained a ready-to-administer dispersion for injection (10 doses of 0.5 mL or 20 doses of 0.25 mL); thus, no prior dilution was required.

#### 2.2.2. Stress Factors Study

The expired Comirnaty™ and Spikevax™ clinical samples were subjected to several stressing conditions: (i) placed in transparent 2 mL glass vials and exposed to accelerated artificial light irradiation (250 W/m^2^) for 24 h in an ageing chamber (Solarbox 3000e RH, Cofomegra, Milan, Italy), (ii) placed in clear 2 mL glass vials and exposed to natural light for 24 h at room temperature (RT), (iii) exposed to mechanical stress by performing several repeated injections (1 and 3) at 300 μL/s using the syringes and the 18 G needles as indicated above, (iv) exposure to mechanical stress by subjecting the samples to manual vigorous shaking for 10 s, and (v) exposed to mechanical stress by subjecting the samples to vibration by vortex at 2400 rpm for 10 s.

The unexpired Comirnaty™ and Spikevax™ samples were subjected to the following stress conditions: (i) exposure to mechanical stress by subjecting the samples to manual vigorous shaking for 10 s and (ii) exposure to mechanical stress by subjecting the samples to vibration by vortex at 2400 rpm for 10 s. This study was conducted in triplicate, with both the unexpired and expired Comirnaty™ and Spikevax™ samples.

#### 2.2.3. Twenty-Four Hour Stability Study

Due to the decision of the Hospital to discontinue the use of Spikevax™, the stability for 24 h was conducted only with Comirnaty™ samples. Then, single doses of 300 µL of Comirnaty™ unexpired dispersions were stored as follows: (i) placed in 1 mL syringes and refrigerated at 2–8 °C protected from daylight, (ii) placed in 1 mL syringes and stored at room temperature (RT) with air conditioning (20 °C) not protected from daylight, (iii) placed in 1 mL syringes and stored at RT without air conditioning (thermal excursion 18–35 °C) not protected from daylight, (iv) placed in 1 mL syringes and stored at −20 °C protected from daylight, and (v) stored in the original vaccine glass vials refrigerated at 2–8 °C protected from daylight. This study was conducted in duplicate.

### 2.3. Visual Characteristics

All vaccine samples were visually inspected to determine colour changes, suspended particulates, turbidity, and gas formation. To this end, samples were visually analysed using the naked eye, and different pictures of the dispersions were taken (Supplementary Data Figure S1) for deeper analysis.

### 2.4. Dynamic Light Scattering (DLS)

The particle size distribution profiles of Comirnaty™ and Spikevax™ clinical dispersions were assessed by DLS. For the analysis, disposable low-volume cuvettes (ZEN0118) for size measurements (IESMAT, Spain) were used. Then, the particles size distribution graphs by volume and intensity, the population hydrodynamic diameter (Dh), the Z-average, and the polydispersity index (PDI) of the samples were evaluated by Photon Correlation Spectroscopy (PCS) using a Dynamic Light Scatterer (DLS, Zetasizer Nano ZS-90, Malvern Panalytical, Malvern, UK), equipped with a backscattered light detector, operating at an angle of 90 ° and a fixed temperature of 25 °C. The results were calculated using the Zetasizer Software ver. 7.13 (Malvern Panalytical, Malvern, UK).

All Comirnaty™ and Spikevax™ unexpired and expired clinical samples were assessed by DLS, that is, samples from stress factors and 24 h stability studies.

### 2.5. Transmission Electron Microscopy (TEM)

This technique was used to characterise the mRNA-LNP morphology and size of the Comirnaty™ and Spikevax™ samples in order to support the DLS data. The samples analysed by TEM were selected, taking into account the DLS results. For Comirnaty™, TEM analyses were performed on unexpired and expired samples. The unexpired ones (control) were subjected to mechanical stress: vortex vibration at 2400 rpm for 10 s and manual vigorous shaking for 10 s; this last sample was 30 days stored at 2–8 °C and reanalysed by TEM. The unstressed expired Comirnaty™ samples were also analysed. In the case of Spikevax™, only expired samples could be analysed; these (control) were subjected to manual vigorous shaking for 10 s and vibration by vortex at 2400 rpm for 10 s.

Dispersion samples were negatively stained. For this, 25 μL of the suspensions were incubated on a grid with supporting film (formvar) for 5 min in a Petri dish. The grids were then washed two times for 1 min with ultrapure water, 2 × 1 min. The contrast was conducted with uranyl acetate (1%) in an aqueous solution for 1 min. Finally, grids were dried on filter paper. Images were obtained using a Zeiss 902 electron microscope (Zeiss, Oberkochen, Germany) operating at an accelerating voltage of 120 kV and observed at a magnification of 50,000.

### 2.6. Data Analysis

Statistical differences in the mean values of Z-average, PDI, and Dh of the populations and their respective percent abundances of all vaccine samples from the stress factors and 24 h stability studies were analysed by one-way analysis of variance (one-way ANOVA), followed by Dunnett’s post hoc test (multiple comparisons with respect to control samples) using GraphPad Prism 7.00 Software. Differences were considered significant at a *p*-value < 0.05.

## 3. Results

### 3.1. Visual Characteristics

Unexpired Comirnaty™ dispersions colour was off-white, whereas Comirnaty™ expired dispersions had an off-white to white colour, which turned more intense the longer the expiration. As for the stresses performed, the turbidity of Comirnaty™ samples increased after being subjected to the specific conditions, mainly by mechanical stresses (pictures shown in Supplementary Data Figure S1). In the case of the Spikevax™ dispersions, the colour was a constant off-white to white colour regardless of the stress condition applied and the expiration date.

### 3.2. Dynamic Light Scattering

#### 3.2.1. Characterisation of Comirnaty™ Clinical Dispersions: A Comparative Study of Unexpired and Expired Samples

**Figure 1:**
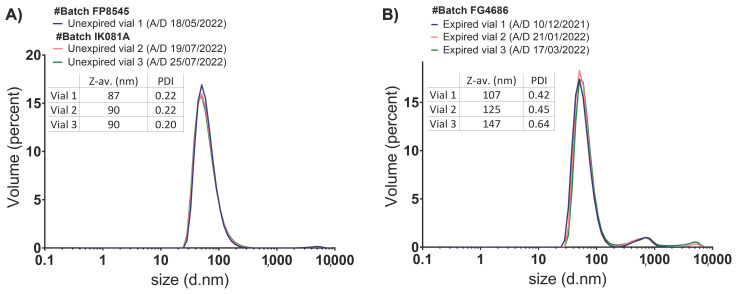
Size distribution graphs by volume of several representative Comirnaty™ samples from (**A**) unexpired and (**B**) expired vials. DLS parameters are included in the table (Z-average and PDI). A/D: Analysis Date; Z-av: Z-average.

The unexpired and expired samples from several Comirnaty™ vials were analysed by DLS to obtain their characteristic particle size distribution profiles. DLS data by volume are shown in Figure 1, whereas intensity data are shown in Supplementary Data Figure S2.

The unexpired Comirnaty™ freshly prepared dispersions from three vials (batches FP8545 and IK081A) were characterised by a main population with a Dh of 60–65 nm that represented over 99% of the total volume in all the vials analysed. A second population of 5000 nm was observed in all the vials, representing less than 1% of the total volume (Figure 1A). Results were similar in these three samples, which had different time left to expiration (two months and several days).

The expired Comirnaty™ samples were also analysed after dilution, and the results are shown in Figure 1B. All vials analysed belonged to the same batch (FG4686) and were 1 month (vial 1), 2 months (vial 2), and 4 months (vial 3) expired by the time of the analyses. The particle size profiles indicated the presence of three clear resolved populations: the most abundant with an average Dh of 60–65 nm accounting for over 90% of the total volume in the samples analysed (vial 1: 94%, vial 2: 92%, and vial 3: 90%), a second population with a Dh of 600–700 nm also detected in all vials on a proportion of 5–10%, and a third population with an average Dh of 5000 nm, only detected in vials 2 and 3 (1.2% and 2.8% by volume, respectively).

These results suggested that the population associated with the individual mRNA-LNPs has an average Dh of 60–65 nm. Then, the other two populations were associated with product-related degradation particles. A gradual decrease in the proportion of the main population in favour of degraded populations in the expired samples, accompanied by increases in Z-average and PDI values over time, was observed (Tables included in Figure 1). The Z-average increased proportionally to expiration times, as did the PDI values. Cumulant analysis confirmed that unexpired samples were relatively monodisperse (PDI around 0.2); on the contrary, expired samples were moderately polydisperse (vial 1 PDI around 0.42 and vial 2 PDI around 0.45) and highly polydisperse (vial 3 PDI around 0.70).

These results clearly indicated an increase in the polydispersity over time in the Comirnaty™ vaccine dispersion by the detection of new larger species.

Particle size distribution graphs by intensity are shown in Supplementary Data Figure S2. As expected, data are similar to the previous ones: a main population is observed in unexpired Comirnaty samples, in addition to the second population at 5000 nm when reported by volume. Expired samples also show a similar size distribution profile with the three size populations. However, intensity data overestimate the high molecular weight species.

#### 3.2.2. Characterisation of Spikevax™ Clinical Dispersions: A Comparative Study of Unexpired and Expired Samples

**Figure 2:**
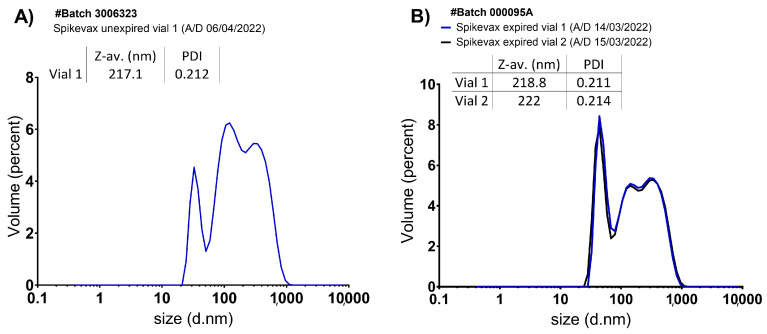
Size distribution graphs by volume of several representative Spikevax™ clinical samples from (**A**) unexpired and (**B**) expired vials. DLS parameters are included in the table (Z-average and PDI). A/D: Analysis Date; Z-av: Z-average.

The unexpired and expired samples from several Spikevax™ vials were analysed by DLS. In Figure 2, DLS data are shown.

Unexpired Spikevax™ dispersion from the vial analysed (batch 3006323) was characterised by three unresolved populations (ranging from 30 nm to 1000 nm) on different percentages, the most abundant with a Dh of 100–200 nm on a 50% approximately (Figure 2A).

Two expired Spikevax™ vials (same expiration times) from the same batch (000095A) were also analysed, both displaying identical size distribution graphs, with three unresolved populations ranging from 30 nm to 1000 nm (Figure 2B) as in the unexpired samples, although the major population observed in this case was the one with a Dh of 30–100 nm, which represented approximately 36% by volume, and the other two populations had Dh of 100–200 nm and 200–1000 nm, respectively. Thus, the population with Dh of 100–200 nm was associated with the single mRNA-LNPs, whereas the other two populations might be explained as product-related degradation. These vials were expired because they had exceeded the 30-day stability at 2–8 °C after withdrawing from the freezer as indicated by the SPC.

Regarding the DLS parameters (Z-average and PDI), no differences were observed between unexpired and expired Spikevax™ samples despite the different size profiles indicated by the graphs (Figure 2). PDI was approximately 0.2 in all samples analysed (moderately monodisperse).

Particle size distribution graphs by intensity are shown in Supplementary Data Figure S2. The fact that the Z-average and PDI of the expired samples do not change in the expired samples with respect to unexpired ones is explained by these graphs: A single polydisperse population is observed, even after stress testing. However, after transforming intensity into volume data, three populations could be detected, and modifications in their relative abundances between expired/unexpired samples and even after stress testing.

#### 3.2.3. Stress Factors Study

To evaluate how handling/mishandling practices may affect the colloidal stability of the dispersions, a set of appropriate stress conditions was checked on the unexpired and expired samples from both vaccines. Expired material already showed starting levels of product-related degradation, i.e., larger species/colour changes (in Comirnaty™) and modifications in the particle size distribution (in Spikevax™), as described in the previous sections. Therefore, evaluating this expired material after subjecting it to stress conditions can be considered the worst-case scenario for the assessment of product robustness against degradation. Comparing the results from the two vaccines analysed under these stress factors allows conclusions to be drawn as to which of the two products is more stable.

**Figure 3:**
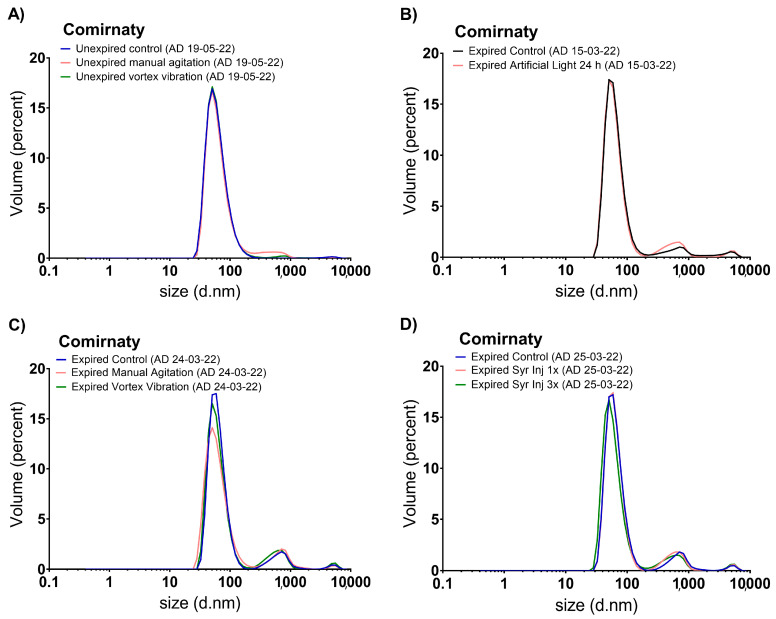
Representative size distribution graphs by volume of several Comirnaty™ unexpired samples subjected to manual shaking and vibration by vortex (**A**) and expired samples subjected to artificial light stress (**B**), manual shaking and vibration by vortex (**C**), and 1 and 3 repeated cycles of injections with syringes (**D**). A/D: Analysis Date.

**Figure 4:**
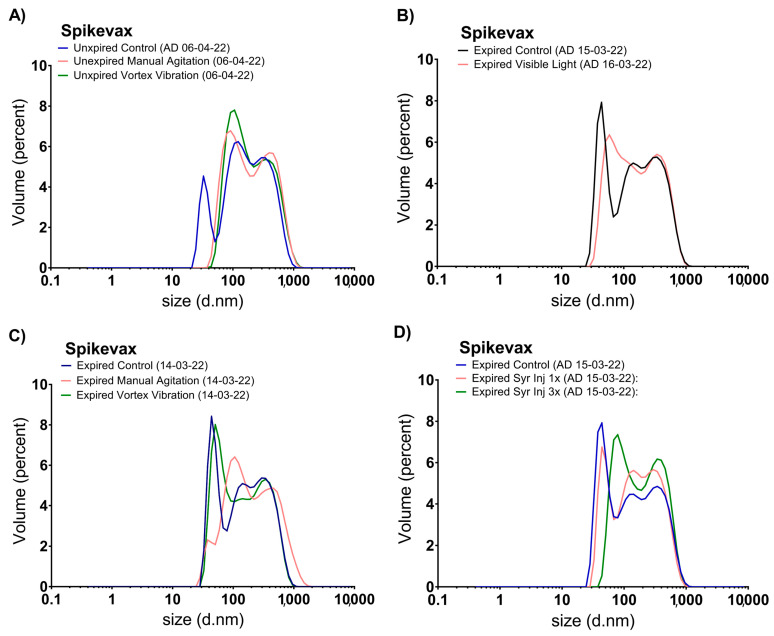
Representative size distribution graphs by volume of Spikevax™ unexpired samples subjected to manual shaking and vibration by vortex (**A**) and expired samples subjected to artificial light stress (**B**), manual shaking and vibration by vortex (**C**), and 1 and 3 repeated cycles of injections with syringes (**D**). A/D: Analysis Date.

Representative size distribution graphs of unexpired and expired samples were established as controls (Figure 3 and Figure 4), in addition to their DLS parameters, expressed as mean ± standard deviation from three independent replicates (Table 2).

In Figure 3A, the size distribution graphs of unexpired Comirnaty™ samples after applying mechanical stress are shown. The control graph indicates a main population with a Dh of 60–65 nm (representing over 99% by volume, Table 2). Mechanical stresses provoked clear degradation (Table 2) in the manually shaken samples (detection of a new polydisperse population with a Dh of 600 ± 60 nm, 5.5% volume) and in the vortex-stressed samples (detection of a new population with a Dh of 1000 ± 200 nm, 1% volume); degradation was proportional to the intensity of the stress. In addition, the volume of the population at 5000 nm decreased in both stress samples (representing a volume inferior to 1% in all cases). DLS parameters reflected these results (Table 2), i.e., the average particle size and the polydispersity increased in the mechanically stressed samples with respect to the control: Z-average from 88 ± 1 nm (control) to 116 ± 8 nm (manual shaking) and to 96 ± 1 nm (vortex stress) and PDI from 0.23 ± 0.01 (control) to 0.38 ± 0.02 (manual shaking) and to 0.26 ± 0.01 (vortex stress). The results were statistically significant for the samples manually shaken (Table 2). However, the results were not statistically significant for the samples vortex stressed.

In Figure 3B–D, graphs from stressed expired Comirnaty™ samples are shown. The control samples show a main population with a Dh of 60–65 nm, representing 88–90% of the total volume. Figure 3B, which shows the artificial light exposure results, can be observed how the populations detected in the expired control samples were kept almost identical after the stress was applied. This was confirmed by the DLS parameters (Table 2). In Figure 3C, concerning mechanical stresses, the populations observed in the stressed samples were similar to the control; however, a significant increase in the abundance of the population with Dh of 600–700 nm was observed when vortex stressed, which also occurred with unexpired samples (Figure 3A). Regarding the DLS parameters, the Z-average values increased (Table 2), indicating an increase in the content of larger species. PDI was kept around 0.7 for all samples, indicating very high polydispersity. In Figure 3D, regarding the samples undergoing cycles of repeated injections, a significant increase in the abundance of the population of 600–700 nm was observed when three cycles were performed, while the DLS parameters were very similar to the expired control with no significant differences, only a slight increase was observed in the Z-average, this indicated that injections caused an increase in the content of larger mRNA-LNP species to a small extent.

In Figure 4A, representative size distribution graphs of unexpired Spikevax™ samples after applying mechanical stresses are shown. In both mechanically stressed samples, only the two larger populations were detected. With regard to the DLS parameters, these were similar to the control with no significant differences, although there were slight increases in the Z-average: from 218 ± 1 nm (control) to 222 ± 3 nm (manual shaking) and the PDI from 0.220 ± 0.003 (control) to 0.23 ± 0.01 (manual shaking) were observed.

In Figure 4B–D, representative graphs from stressed expired Spikevax™ samples are shown. In Figure 4B, the size distribution graphs of samples light stressed were slightly different to the control ones; the main population with a Dh of 30–100 nm slightly increased its size. However, the DLS parameters were not modified (Table 2). Figure 4C shows that the vortex did not affect the size distribution, whereas an increase in the average particle size was observed when manually shaken. Again, with regard to the DLS parameters, no significant differences were detected, although a slight increase in the Z-average after manual shaking was observed (Table 2), as occurred with unexpired samples. Figure 4D indicates that one injection cycle did not promote changes in the vaccine dispersion; however, after three repeated cycles, the main population (Dh 30–100 nm) disappeared, and a new population with a higher size (40–200 nm) was detected; the DLS parameters were not altered in any of the samples despite these observed modifications in the size distribution graphs (Table 2).

#### 3.2.4. Comirnaty™ 24-h Stability Study (Unexpired Samples)

**Figure 5:**
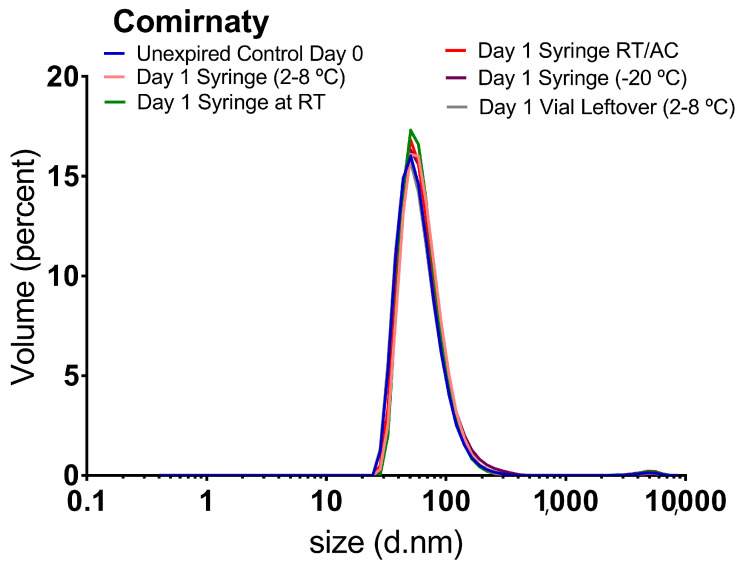
Representative size distribution graphs by volume of Comirnaty™ clinical samples at day 0 (unexpired control) and after storage under different clinical conditions for 24 h. RT: Room Temperature; AC: Air Conditioning.

As indicated in the Materials and Methods section, the 24 h stability study was performed just on Comirnaty™ due to the impossibility of obtaining units of Spikevax™ as a result of the change in the purchase of the COVID-19 vaccines by the Hospital Pharmacy Management Unit. This study aimed to evaluate the stability of individual doses of Comirnaty™ prepared under clinical conditions. Thus, they were stored in 1 mL syringes under different environmental conditions (Section 2.2.3), intending to determine how different daily hospital practises may affect the physical stability of the dispersions. Representative size distribution graph of an unexpired Comirnaty™ freshly prepared sample (Figure 5), in addition to the DLS parameters, expressed as mean ± standard deviation from two independent replicates (Table 3), were established as control.

The results for Comirnaty™ control samples were consistent with the previously characterised control samples (Section 3.2.1). The size distribution graph (Figure 5) showed the main population at 60–65 nm, representing over 99% of the total volume and the larger population of 4500–5000 nm, representing a rough 1%. After comparing the control and 24 h stored samples, no meaningful changes were observed in the size distribution graphs for any tested conditions. DLS parameters remained unaffected as well in all samples except for the case of those stored in syringes at −20 °C; in this case, the Z average increased significantly from 89.3 nm ± 0.9 nm to 99.2 nm ± 0.8 nm and PDI from 0.20 to 0.23 nm, indicating increase in the average particle size (Table 3).

To confirm this last, the samples stored at −20 °C were again subjected to a second freeze/thaw cycle, and it was corroborated that freezing/thawing promotes increases in the Z-average and PDI parameters (112 nm and 0.25, respectively after the second F/T cycle), concluding that freeze/thaw cycles increase the content of larger species. For all the other conditions tested, Comirnaty™ clinical dispersions were demonstrated to be stable regardless of the container, that is, syringes or vials; the temperature, that is, room temperature with temperature excursions (18–35 °C) or refrigerated between 2 and 8 °C; and the exposition or not to the daylight.

### 3.3. Transmission Electron Microscopy (TEM) Analysis

To deeply characterise the mRNA-LNPs’ physical properties, their size and morphology were evaluated by TEM; this allowed a comprehensive physical characterisation that also served for comparative purposes among the two marketed vaccines and also both methodologies (DLS and TEM). Samples from Comirnaty™ and Spikevax™ were again subjected to several stress conditions that were selected, taking into account the DLS results from Section 3.2.3, where physical degradation was demonstrated.

**Figure 6:**
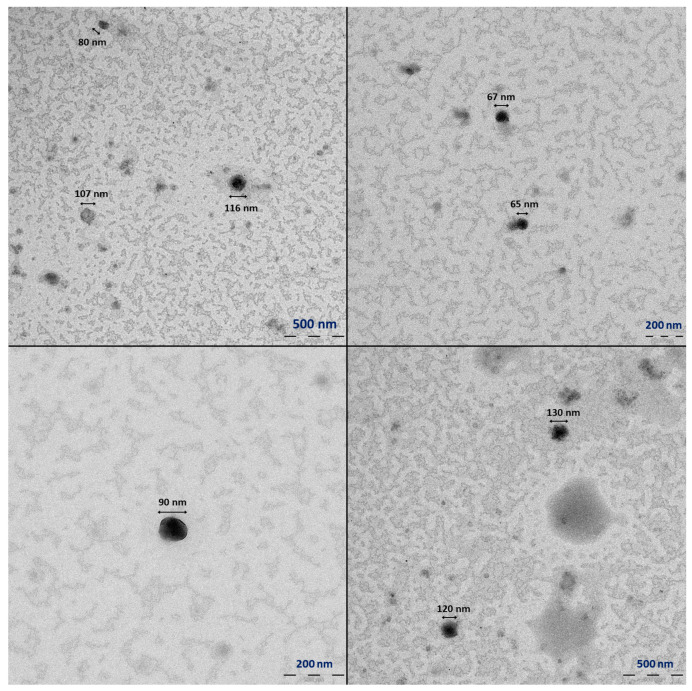
Representative Comirnaty LNPs from a single unexpired control sample: micrographs taken at different levels of amplification and positions.

**Figure 7:**
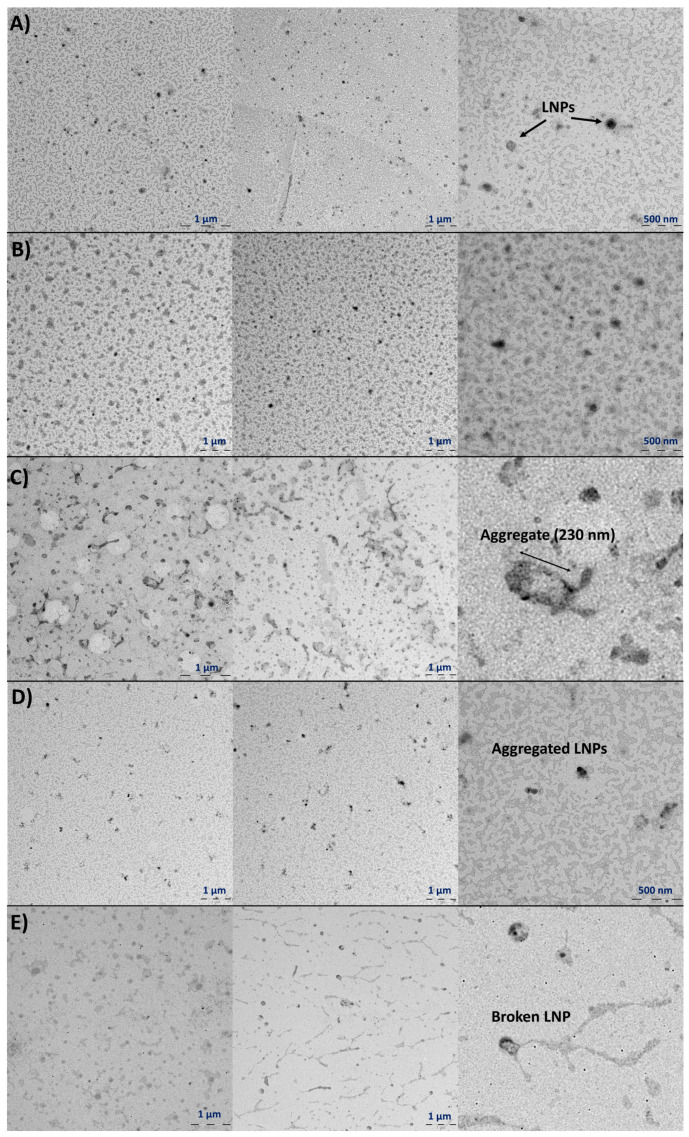
Representative TEM micrographs of (**A**) unexpired Comirnaty™ sample (control) at day 0 of preparation, (**B**) expired Comirnaty™ sample, (**C**) unexpired Comirnaty™ sample subjected to manual shaking, (**D**) sample C aged for 30 days at 2–8 °C, and (**E**) unexpired Comirnaty™ sample subjected to vortex vibration.

Comirnaty™ TEM micrographs were characterised by black stained particles on a grey background that were attributed to the mRNA-LNPs (Figure 6). The unexpired control samples show nanoparticles with homogenous and regular round shapes with average diameters of 90–100 nm, which corroborates the results obtained by DLS (Section 3.2.1). Micrographs from expired Comirnaty™ samples indicated an increase in the average particle size; amorphous stained particles were observed in addition to spherical ones. Expiration induces aggregation of the nanoparticles, as illustrated in Figure 7B.

With regard to stress testing (Figure 7), changes in the size and morphology of the nanoparticles were observed. The unexpired control samples subjected to manual shaking and vibration by vortex contain degraded particles. Long and dense regions of stained material were observed, which indicated that mechanical stressors induced breakage/aggregation of the mRNA-LNPs (Figure 7C, manual shaking and Figure 7E, vortex vibration). The degradation effect was more evident in manually shaken samples. Micrograph from the sample stored for 30 days at 2–8 °C after manual shaking (Figure 7D) showed broken nanoparticles and also self-assemblies.

Additionally, DLS parameters were registered for these specific samples and are shown in Table 4. After expiration (8 months), the average particle size was twice the control value, while the polydispersity increased significantly to 0.7; this very high polydispersity is explained by the identification of particles with a wide range of sizes and morphologies (Figure 7B). In the case of unexpired samples subjected to manual shaking, significant increases in these parameters were also observed, although less pronounced than in the expired sample. Aggregates and amorphous particles were detected by TEM; however, many spherical particles still could be observed. After storing this sample at 4 °C for 30 days, the DLS parameters were kept, which indicated that modifications were irreversible. Finally, vortex stress induces similar—although less aggressive—modifications than manual shaking.

**Figure 8:**
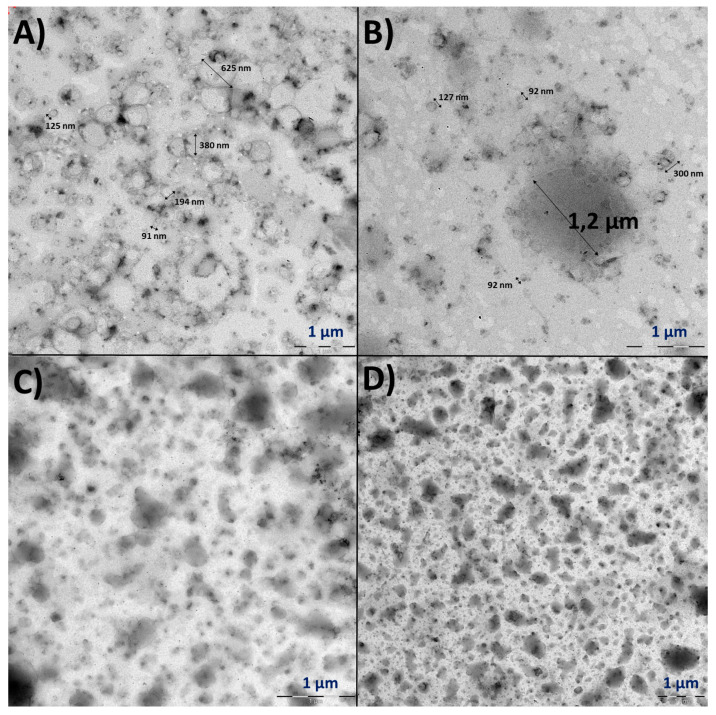
Representative TEM micrographs of (**A**) expired Spikevax™ control sample, (**B**) expired Spikevax™ sample subjected to manual shaking, and (**C**,**D**) expired Spikevax™ sample subjected to vortex vibration.

The expired Spikevax™ TEM micrographs were characterised by many round particles of different sizes (Figure 8). Figure 8A illustrates spherical polydisperse particles in a wide range of diameters (up to 625 nm), which agrees with DLS results. As for the sample subjected to manual shaking, similar results were obtained; round particles from 90 nm to 1.2 µm can be observed in this case (Figure 8B); the 1.2 µm particle can be associated with aggregates. The increase in the size of the third population observed by DLS after applying manual shaking can explain this fact (Figure 4C). Finally, the vortex-stressed sample (Figure 8C,D) also contained polydisperse particles with different sizes and morphologies; round particles can be distinguished with an approximate size of 70–200 nm, in addition to bigger ones that, in some cases, have an approximate diameter of 1 µm.

## 4. Discussion

In this study, a comparative stability study of the main produced and administered vaccines worldwide, i.e., Comirnaty™ and Spikevax™, was conducted regarding the size and homogeneity of the mRNA-LPNs. These parameters, in addition to being relevant for reproducible manufacturing [16], also influence potency as observed in siRNA-LNP model systems for LNP size [17]. Therefore, modifications to these parameters are straightforwardly related to the stability of the vaccines. Then, the particulate characteristics of the two vaccine dispersions analysed by DLS and TEM are shown here. DLS is a qualitative stability indicating the method used for quality control in the manufacturing of the two vaccines (LNP size and polydispersity) [18,19]. TEM was used to better interpret and support the DLS results. The present stability study upon preparation/storage and after subjection to different mild and strong stress conditions shed light on the stability of these novel mRNA-LNP vaccines, for which there is scarce literature in this regard.

Comirnaty™ and Spikevax™ vaccines were developed in parallel and mass-produced during the COVID-19 pandemic at record speed, having demonstrated safety and efficacy worldwide [20,21]. However, a major stumbling block was the stability of this novel vaccine technology [10]. These two mRNA-LNP vaccines are similar, with some differences regarding lipid composition, formulation, and presentation [4,11,15] 

The characterisation of the vaccine dispersions indicated consistent results for the size of unexpired Comirnaty™ nanoparticles compared to existing literature [12,13], and also for the expired samples [14]. However, there are no published data regarding the size of Spikevax™ nanoparticles as far as we know, although there is literature stating sizes of 80–100 nm [1]. Our results showed that the dispersions differed significantly in their size distribution profiles; the Z-average of the mRNA-LNPs was higher in the Spikevax™ samples, indicating a larger average size of the mRNA-LNPs. However, both vaccines had similar PDI values, which can be explained by the detection of a population in the Comirnaty™ samples of approximately 5000 µm (≤1% of the total volume). On the other hand, DLS data confirmed differences between unexpired and expired material from both vaccines. In the case of Comirnaty™, unexpired samples were significantly different to the expired ones in regards to solution colour, size distributions and DLS parameters; in the case of Spikevax™, although significant differences were not found in the DLS parameters, size distributions were remarkably different in expired samples compared to unexpired ones.

The stress factors study was aimed at assessing the in-use stability of clinical samples of the vaccines following their preparation. For this purpose, different relevant stressors were applied to samples from both vaccines. The results show that Comirnaty™ has a natural likelihood to undergo aggregation, giving rise to two new particle populations by DLS in addition to the main population associated with the individual mRNA-LNPs. The physical stability of Comirnaty™ was mainly affected by mechanical stresses, as previously described and discussed [12], and was not affected by the effects of light. Avoiding quick dilutions, vigorous mixing, and accidental syringe/vial drops is paramount to ensure colloidal stability. In contrast, Spikevax™ dispersions showed a more robust behaviour against stresses: size distributions were not easily readable, as the three unresolved populations ranging from 30 to 1000 nm were present in unexpired, expired, and stressed samples. However, mechanical stresses seemed to favour the formation of larger mRNA-LNP populations, as shown by DLS; in fact, the Z-average values increased slightly in manually shaken unexpired and expired samples. Recently, Grau et al. [22], assessed the stability of the mRNA molecules from Pfizer-BioNTech and Moderna samples in syringes under movement conditions, simulating the transport of reconstituted syringes by road to the vaccination centres; here, they demonstrated the stability of the mRNAs from both vaccines under swing agitation conditions during 3 h. However, they reported mRNA degradation in both vaccines after vortex stress.

The Comirnaty™ 24 h study aimed to evaluate the stability of Comirnaty™ after repackaging and storage of single doses in 1 mL plastic syringes. The results indicated a high stability of the dispersions under most of the storage conditions checked. This was also corroborated by other researchers [12]; they demonstrated stability of the dispersions for 24 h stored refrigerated (2–8 °C) and at room temperature (25 °C), and 5 h at 40 °C, regardless of the packaging material, i.e., polypropylene or carbonate syringes. In our study, Comirnaty™ clinical dispersions were affected by storage at −20 °C for 24 h (having undergone 1 F/T cycle). Freezing is one of the most widely used methods for long-term storage of many types of nanoparticles and is harmless when cryoprotectants are ensured [23]. However, it can alter the stability of NPs by crystallisation and vacuum dehydration. In fact, aggregation of lipid nanoparticles was described mainly due to the freeze/thaw [24]. The DLS results here indicated a clear increase in larger species after thawing from −20 °C to room temperature.

To better explore the mRNA-LNPs, TEM was proposed as an orthogonal method to DLS, allowing for a full characterisation of the mRNA-LNPs physical characteristics, concluding that the results obtained by both techniques were comparable. Indeed, these are widely used techniques to study the size properties of NPs. Some articles provide comparative studies using both techniques to determine the size of NPs [25,26]. The TEM micrograph of the Comirnaty™ control sample (unexpired) indicated spherical monodisperse particles, whose sizes were in agreement with DLS size results. Once the samples expired, an increase in the DLS parameters was associated with an increase in the average particle size by TEM and also with loss of sphericality. As for the stress tests, manual shaking and vortex stresses caused a clear increase in the DLS parameters and were associated with breakage and aggregation of the mRNA-LNPs. Although these stress conditions checked are strong and unlikely to occur in a realistic situation, the results highlight the way mechanical stresses might induce irreversible degradation of the nanoparticles. Indeed, milder stress conditions have indicated starting degradation by DLS, such as Comirnaty™ samples undergoing freeze/thaw cycles or repeated cycles of aspiration cycles with syringes. The TEM results from Spikevax™ were more complex to analyse; a great number of particles and a wide range of sizes and morphologies were observed in all the samples. Nevertheless, it can be concluded that Spikevax™ samples were far more polydisperse than Comirnaty™.

Preserving the physical integrity of LNPs in mRNA vaccines is of paramount importance as they serve to protect the mRNA from release, thus preventing the action of endonucleases and increasing bioavailability in vivo. Not only can vaccine efficacy be jeopardised by mRNA degradation, but patient safety can also be an issue if mRNA-LNPs aggregate, as they could elicit undesirable immune reactions [27]. In this way, studies indicate that ideal nanoparticle sizes for drug delivery range between 10 nm and 1000 nm [28]. In the case of mRNA vaccines, uptake by antibody presenting cells is crucial being demonstrated ideal sizes to be 80–100 nm [29], whereas smaller LNP sizes favour recognition by dendritic cells and bigger ones favour recognition by macrophages [30].

## 5. Concluding Remarks

We show remarkable stability data for Comirnaty™ (Pfizer-BioNTech) and Spikevax™ (Moderna) COVID-19 vaccines after preparation/storage and stress testing. Although both products are based on the same mRNA-LNP technology, the results presented here demonstrate that they are characterised by different particle size profiles, as well as behaviours against the different handling/stress conditions. Comirnaty™ was shown to be mostly sensitive to mechanical stresses and freeze/thaw cycles from −20 °C to room temperature, less affected by syringes injection cycles and not affected at all by the effects of light. On the other hand, Spikevax™ samples were proven to be stable under most of the stress conditions tested with regard to DLS parameters, although changes in size distribution profiles were observed, particularly in samples subjected to mechanical stress. These data will be meaningful for healthcare professionals in hospitals and healthcare facilities, for the results clearly highlight the deleterious effects of improper manipulation on the sizes of the particulate of the vaccine dispersions, eventually affecting potency. This study was not intended to assess the chemical stability of the mRNA molecule, which will be addressed in future research using high-resolution mass spectrometry analyses. The assessment of the mRNA’s chemical and physical properties is also fundamental, as modifications of these properties might occur inside the protective nanoparticle, going unnoticed. On the other hand, this study also provides valuable knowledge on the different behaviour of mRNA-LNPs and, for the first time, in-use stability for the Spikevax™ vaccine.

## 6. Limitations of the Study

One of the main limitations of this study was the limited availability of Spikevax™ vials, especially unexpired Spikevax™ ones. In fact, the supply of these vials was stopped midway in the investigation. Thus, more conclusions could be drawn regarding the stability of Comirnaty™ than Spikevax™. Another aspect to take into account is that the unexpired Spikevax™ vial analysed was proximate to its end-to-use date, which might explain the similar results obtained between both expired and unexpired samples. Despite this, it was possible to draw consistent conclusions regarding the in-use stability of both products since several expired vials from both vaccines were available.

## Figures and Tables

**Table 1:** Comirnaty™ and Spikevax™ batches used in the study.

Vaccine	Expired Batches	Unexpired Batches
Comirnaty™	FG4686 (Exp. 11/2021) ^1^	FP8545 (Exp. 07/2022) ^1^ IK081A (Exp. 07/2022) ^2^
Spikevax™	000095A (Exp. 05/2022) ^1,3^	3006323 (Exp. 05/2022) ^1^

^1^ Vials from these batches were used in the forced degradation studies; ^2^ Vials from this batch were used in the 24 h stability study; ^3^ Exceeded for more than 30 days after defrosting.

**Table 2:** Stability of Comirnaty™ and Spikevax™ clinical samples after stress testing: assessment of Z-average and PDI by DLS.

	Parameters	Stress Test	Z-Average (nm)	PDI	Population 1 (Volume)		Population 2 (Volume)		Population 3 (Volume)	
Vaccines					D_h_ ± SD (nm)	vol ± SD (%)	D_h_ ± SD (nm)	vol ± SD (%)	D_h_ ± SD (nm)	vol ± SD (%)
Pfizer-BioNTech (Comirnaty™)		Control 1 (E)	147 ± 3	0.69 ± 0.04	64 ± 1	89.6 ± 0.7	700 ± 60	9 ± 1	4800 ± 600	2 ± 1
		Artificial light 24 h	146 ± 2	0.70 ± 0.04	63 ± 2	87.9 ± 0.3	600 ± 50	10.3	5000 ± 100	1.8 ± 0.3
		Control 2 (E)	149 ± 4	0.70 ± 0.04	62 ± 2	88.60 ± 0.06	650 ± 40	9.7 ± 0.6	5100 ± 100	1.7 ± 0.6
		Manual shaking (E)	170 ± 20	0.70 ± 0.10	61 ± 2	87.2 ± 0.3	680 ± 70	11.6 ± 0.9	5090 ± 30	1.3 ± 0.6
		Vortex vibration (E)	165 ± 7	0.74 ± 0.04	63 ± 1	86 ± 1	400 ± 300	13 ± 1 *	5100 ± 200	1.6 ± 0.3
		Control 3 (E)	147.1 ± 0.6	0.71 ± 0.03	63 ± 2	88.9 ± 0.6	700 ± 10	9.3 ± 0.4	4990 ± 60	1.8 ± 0.6
		Syringe injection (1x) (E)	149 ± 3	0.73 ± 0.01	62.7 ± 0.5	87.7 ± 0.7	620 ± 20	10.5 ± 0.8	5000 ± 100	1.7 ± 0.2
		Syringe injection (3x) (E)	156 ± 6	0.66 ± 0.01	63 ± 2	86.9 ± 0.8	610 ± 30	12 ± 1 *	5100 ± 40	1.5 ± 0.4
		Control 4 (NE)	88 ± 1	0.23 ± 0.01	63.0 ± 0.9	99.40 ± 0.06	NP	NP	4400 ± 200	0.630 ± 0.06
		Manual shaking (NE)	116 ± 8 *	0.38 ± 0.02 *	65.1 ± 0.5	94.4 ± 0.3 *	600 ± 60	5.5 ± 0.3	4873	0.1 ± 0.2
		Vortex vibration (NE)	96 ± 1	0.26 ± 0.01	65.2 ± 0.9	98.5 ± 0.6	1000 ± 200	1 ± 1	4571	0.3 ± 0.5
Moderna (Spikevax™)		Control 1 (E)	220 ± 2	0.22 ± 0.01						
		Natural light 24 h (E)	215 ± 2	0.22 ± 0.01						
		Control 2 (E)	221 ± 4	0.21 ± 0.01						
		Manual shaking (E)	222 ± 3	0.23 ± 0.01						
		Vortex vibration (E)	220 ± 4	0.22 ± 0.01						
		Control 3 (E)	220 ± 2	0.22 ± 0.01						
		Syringe injection (1x) (E)	220 ± 2	0.22 ± 0.01						
		Syringe injection (3x) (E)	219 ± 3	0.22 ± 0.01						
		Control 4 (NE)	218 ± 1	0.220 ± 0.003						
		Manual shaking (NE)	222 ± 3	0.23 ± 0.01						
		Vortex vibration (NE)	216 ± 2	0.21 ± 0.01						

Data are reported as mean ± Standard Deviation (SD) from 3 independent replicates; * Indicate significant differences with respect to control; E: Expired; NE: Unexpired; NP: Not Present; PDI: Polydispersity Index.

**Table 3:** Twenty-four h stability of Comirnaty™ clinical samples after storage in syringes: assessment of Z-average, PDI, and D_h_ of populations by DLS.

	Parameters	Storage Condition	Z-Average (nm)	PDI	Population 1		Population 2		Population 3	
Vaccine					D_h_ ± SD (nm)	vol ± SD (%)	D_h_ ± SD (nm)	vol ± SD (%)	D_h_ ± SD (nm)	vol ± SD (%)
Pfizer-BioNTech (Comirnaty™)		Control (unexpired)	89.3 ± 0.9	0.20	66 ± 1	99.7 ± 0.1	NP	NP	4900 ± 100	0.4 ± 0.1
		Syringe 24 h 2–8 °C	90 ± 1	0.20	65.7 ± 0.5	99.7 ± 0.1	NP	NP	4600 ± 400	0.3 ± 0.1
		Syringe 24 h RT/AC (20 °C)	91 ± 1	0.22	67 ± 1	99.4	NP	NP	4810 ± 90	0.60
		Syringe 24 h RT	91 ± 1	0.21	65.5 ± 0.7	99.4 ± 0.1	NP	NP	4740 ± 80	0.6 ± 0.1
		Syringe 24 h (−20 °C)	99.2 ± 0.8 *	0.23 *	72 ± 2	99.1 ± 0.3	889.8	0.2	4400 ± 200	0.8 ± 0.1
		Vial leftover 24 h (2–8 °C)	91.6 ± 0.1	0.22	65 ± 3	99.5 ± 0.3	887.1	0.1	4500 ± 700	0.5 ± 0.2

Data are reported as mean ± Standard Deviation (SD) from 2 independent replicates; * Indicate significant differences with respect to control; indicates the population was present in one replicate; AC: Air Conditioning; Exp: Expired; NP: Not Present; RT: Room Temperature.

**Table 4:** Assessment of the DLS parameters of Comirnaty™ samples analysed by TEM.

	Parameters	Stress Test	Z-Average (nm)	PDI
Vaccines				
Pfizer-BioNTech (Comirnaty™)		Unexpired	88.7 ± 0.9	0.20 ± 0.02
		Manual shaking	116 ± 8	0.38 ± 0.02
		Manual shaking (30 days at 4 °C)	112 ± 1	0.39 ± 0.01
		Vortex vibration	96 ± 1	0.26 ± 0.01
		Expired	157 ± 6	0.71 ± 0.06

## Data Availability

Data will be provided on request.

## Supplementary Materials

The following supporting information can be downloaded at: https://www.mdpi.com/article/10.3390/vaccines11111635/s1, Figure S1: Photographs of expired (A) and non-expired (B) Comirnaty vials; expired (C) and non-expired (D) Spikevax vials.; Figure S2: Representative particle size distribution graphs by intensity of unexpired Comirnaty™ (A), unexpired Spikevax™ (B), expired Comirnaty™ (C) and expired Spikevax™ (D) samples subjected to mechanical stress.
