# Supplementary material for: Analysing the In-Use Stability of mRNA-LNP COVID-19 Vaccines Comirnaty™ (Pfizer) and Spikevax™ (Moderna): A Comparative Study of the Particulate
Source: Vaccines (Basel). 2023 Oct 25;11(11):1635. doi: 10.3390/vaccines11111635 (PMC10675537; doi:10.3390/vaccines11111635)
Supplement: Supplementary file 1 [file vaccines-11-01635-s001.zip › vaccines-2616399-supplementary.pdf]

Supplementary Data Figure S1

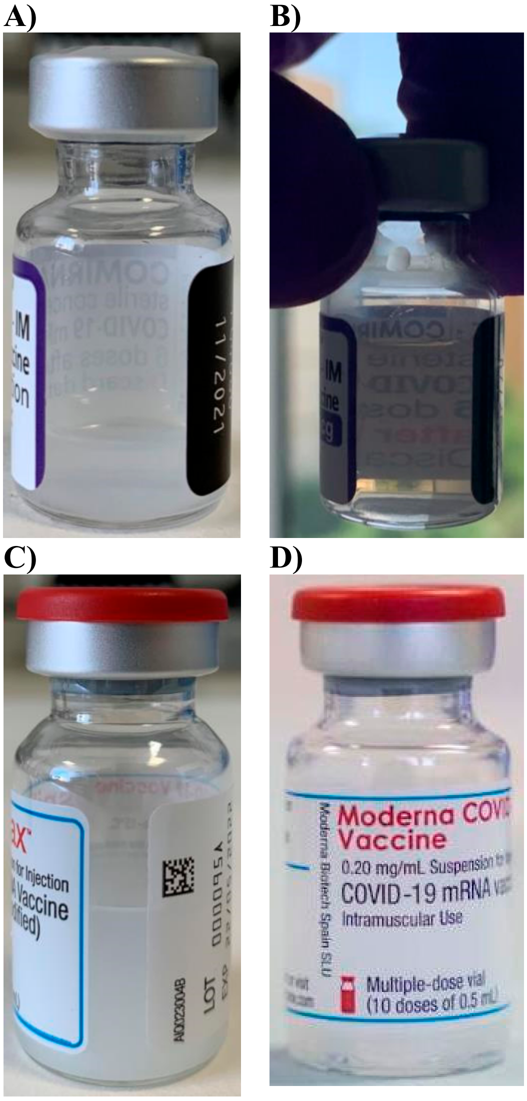

Supplementary Data Figure S1. Photographs of expired (**A**) and non-expired (**B**) Comirnaty vials; expired (**C**) and non-expired (**D**) Spikevax vials.

Supplementary Data Figure S2

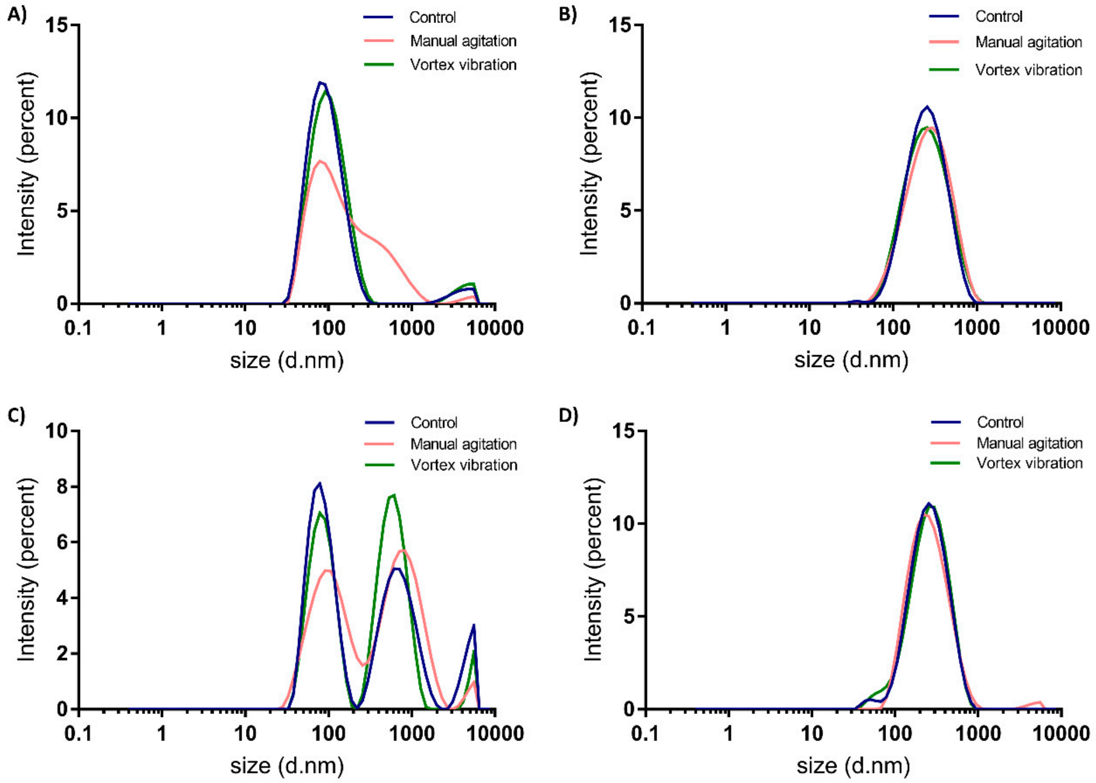

Supplementary Data Figure S2. Representative particle size distribution graphs by intensity of unexpired Comirnaty™ (**A**), unexpired Spikevax™ (**B**), expired Comirnaty™ (**C**) and expired Spikevax™ (**D**) samples subjected to mechanical stress.
